# Metabolic Syndrome Among Overweight and Obese Children Visiting Tertiary Care Hospital of Jharkhand: A Cross-Sectional Study

**DOI:** 10.7759/cureus.116684

**Published:** 2026-09-21

**Authors:** Mandeep Kaur, Sarthak Das, Vinayagamoorthy Venugopal, Saroj K Tripathy, Ehtesham Ansari, Soumi Kundu, Archana Malik

**Affiliations:** 1 Pediatrics, All India Institute of Medical Sciences, Jharkhand, IND; 2 Community and Family Medicine, All India Institute of Medical Sciences, Jharkhand, IND; 3 Pulmonary Medicine, All India Institute of Medical Sciences, Jharkhand, IND

**Keywords:** childhood obesity, cross-sectional study, dyslipidemia, india, jharkhand, metabolic syndrome, overweight, risk factors

## Abstract

Background: Childhood obesity is a growing public health challenge and is closely linked to metabolic syndrome, a cluster of cardiometabolic abnormalities that predispose to type 2 diabetes and cardiovascular disease. Data on the prevalence and determinants of metabolic syndrome among Indian children, particularly in underserved regions, remain limited.

Objective: The aim of the study is to estimate the prevalence and predictors of metabolic syndrome among overweight and obese children attending a tertiary care hospital in Jharkhand, India.

Methods: A hospital-based cross-sectional study was conducted between January 2024 and March 2025 among 250 overweight and obese children aged 5-15 years at AIIMS Deoghar. Anthropometric measures, blood pressure, and fasting biochemical parameters (glucose, triglycerides, high-density lipoprotein, low-density lipoprotein, and total cholesterol) were assessed. Metabolic syndrome was defined using the Indian Academy of Pediatrics (IAP) criteria. Statistical analyses included chi-square tests, t-tests, and logistic regression to determine independent predictors.

Results: The prevalence of metabolic syndrome was 38% (n=95; 95% CI: 31.9-44.1). Significant risk factors included family history of obesity (OR: 1.7, 95% CI: 1.22-2.48), excess junk food intake (OR: 1.58, 95% CI: 1.18-2.11), abnormal developmental history (OR: 2.07, 95% CI: 1.39-3.08), low physical activity (<1 hour/day; OR: 1.67, 95% CI: 1.07-2.59), screen time >2 hours/day (OR: 1.44, 95% CI: 1.01-2.07), and birth weight <2.5 kg (adjusted OR: 1.43, 95% CI: 1.09-1.88). Protective factors included exclusive breastfeeding for six months (p<0.001). No significant association was observed with gender or parental education.

Conclusion: Metabolic syndrome is highly prevalent among overweight and obese children in Jharkhand, with both lifestyle-related (diet, physical activity, and screen time) and perinatal (low birth weight and maternal diabetes/hypertension) factors contributing. Early interventions targeting modifiable risk factors through lifestyle modification and health education are essential to reduce long-term cardiometabolic risks in this population.

## Introduction

Childhood obesity is now a global epidemic in developed nations. It leads to numerous health problems, raising both illness rates and early mortality [1]. The drivers of obesity during childhood and youth are varied and complex [2]. Worldwide rates of pediatric obesity have risen sharply in recent decades. The fastest increase has occurred in low- and middle-income countries. This rapid rise is contributed to by sedentary lifestyles, higher incomes, ongoing urbanization, and broader access to calorie-dense, processed foods.

​Childhood overweight and obesity are well-established precursors to cardiometabolic disease. They significantly increase the likelihood of developing metabolic syndrome at an early age [3]. Metabolic syndrome is a cluster of cardiometabolic abnormalities. These include central obesity, dyslipidemia, high blood pressure, and elevated blood glucose. Together, they strongly increase the risk of adult type 2 diabetes and cardiovascular disease. Over the past two decades, rising pediatric obesity has driven an escalating global burden of metabolic syndrome in young people [4]. General population surveys show metabolic syndrome in approximately 3.6% of all children, 11% of overweight youth, and 29.2% of adolescents [5,6]. However, the prevalence is substantially higher among children with obesity [5,6]. Key lifestyle factors contribute directly to this risk -- sedentary habits, prolonged screen time, skipped meals, and inadequate sleep are all closely linked to metabolic syndrome even in pre-adolescents [7,8].

South Asian populations, including Indians, develop cardiometabolic diseases at lower body mass index (BMI) thresholds and younger ages. National estimates indicate an overall metabolic syndrome prevalence of 5.2% among adolescents. This burden is notably higher in higher socioeconomic status, urban population presenting with central obesity, elevated blood pressure, and dyslipidemia [9,10]. While general lifestyle factors are commonly evaluated, cross-sectional studies frequently omit data on perinatal insults, specifically low birth weight and distinct maternal and paternal cardiometabolic clustering. Although physical inactivity and caloric surplus are established correlates of pediatric obesity, conventional risk assessments rarely measure screen time exposure and low birth weight. Consequently, investigating the regional rise in pediatric metabolic syndrome is critically important.

We conducted this study to assess the prevalence and associated factors of metabolic syndrome among overweight and obese children in Jharkhand.

## Materials and methods

Study design

This was an observational cross-sectional hospital-based study.

Inclusion criteria

Overweight and obese children aged 5-15 years who visited the OPD/IPD of the All India Institute of Medical Sciences, Deoghar, from May 2024 to May 2025 were included.

Exclusion criteria

Overweight and obese children with unstable vitals or previously diagnosed with comorbidities, with underlying chronic medical conditions, including genetic syndromes, secondary obesity from endocrinopathies and other causes (except exogenous nutritional factor), familial or primary hypercholesterolemia, inherited inborn error of metabolism, or any liver, kidney, respiratory, neurological, gastrointestinal, genitourinary and heart diseases, were excluded from this study.

Sampling strategy

Participants were recruited using a convenience sampling strategy, whereby eligible overweight and obese children attending the pediatric outpatient and inpatient department during the study period were consecutively enrolled until the required sample size was achieved.

Sample size calculation

The sample size was calculated to be 250 using the formula N = (Z₁−α/₂)²p(1−p)/d², where Z₁−α/₂ represents the standard normal deviate at a 95% confidence level, and p represents the previously reported proportion of metabolic syndrome (3.6%) among overweight and obese children. The calculation used 15% relative precision, 95% confidence limits, and a 10% allowance for non-response [11].

Ethical clearance

After obtaining ethical clearance from the Institutional Ethics Committee (IEC No. 2024-301-IND-04), written informed consent was obtained from the parents/guardians and assent from eligible children. The included cases were subsequently approached, clinically evaluated, and managed according to the guidelines recommended by the Indian Academy of Pediatrics (IAP) and the European Society for Paediatric Endocrinology [12,13].

Study outcomes

Clinical history and anthropometric measurements such as body weight, height, BMI, and waist circumference (WC) were recorded.

Anthropometry and vital measurement

Body weight was measured in kilograms up to one decimal place using a digital weighing scale, and height was measured up to one decimal place using a standard stadiometer. BMI was expressed in kg/m^2^ by dividing weight (in kilograms) by the square of the height (in meters). WC was measured using a measuring tape, midway between the lowest ribs and the iliac crest, in the standing position [14]. Blood pressure was measured using a digital blood pressure machine, recorded manually, and interpreted using a BP percentile chart [15].

Blood parameter measurement

A fasting venous blood sample of 2 mL was collected under aseptic conditions after taking informed written consent, and the following biochemical parameter values were documented, including fasting blood glucose, triglycerides (TGs), total cholesterol, high-density lipoprotein (HDL), and low-density lipoprotein (LDL). The samples were processed in the central biochemistry laboratory using the dry chemical calorimetric method on an automated Vitros 5600 instrument. The LDL-cholesterol level was calculated by the Friedewald formula for TG concentrations up to 250 mg/dL in the laboratory itself. For TG values greater than 250 mg/dL, it was directly assessed in Vitros 5600 [16].

Statistical analysis

Data were entered in Microsoft Excel (Microsoft Corp., Redmond, WA, USA) and analyzed using SPSS software version 24.0 (IBM Corp., Armonk, NY). Descriptions of categorical study variables were calculated as percentages, and those of continuous variables as mean and standard deviation. The proportion of metabolic syndrome among overweight and obese children was calculated and reported with a 95% confidence interval. Parametric analysis was performed using one-way analysis of variance and Student’s t-test. Multiple regression analysis was used to evaluate the independent predictors of metabolic syndrome in these subjects. All tests were two-tailed, and p-values < 0.05 were considered statistically significant.

Operational definition

Metabolic Syndrome

It was defined as WC ≥90th percentile for age/gender/Indian ethnicity (as per IAP reference charts) plus any two or more of the following components: (1) serum TGs ≥150 mg/dL, (2) HDL-cholesterol <40 mg/dL, (3) fasting plasma glucose ≥100 mg/dL, and (4) blood pressure ≥130/85 mmHg or ≥95th percentile for age/height/sex [17].

Overweight

Overweight was defined as a BMI exceeding 23 kg/m² in adults [18].

Obesity

Obesity was defined as a BMI >27 kg/m², based on the Indian cut-offs derived from the IAP 2015 BMI-for-age charts, which were developed using adult BMI criteria [14,18].

*Screen Time* 

Screen time was defined according to the IAP guidelines, and a threshold of >2 hours/day was adopted for the age groups included in our study. Parents were instructed to record their child’s daily mobile phone usage using either a diary method or the Digital Wellbeing/Screen Time feature available on the mobile phone [18]. 

## Results

A total of 250 children aged 5 to 15 years who were overweight and obese participated in this study. The prevalence of metabolic syndrome was 38% (95 out of 250), with a confidence interval ranging from 31.9 to 44.1 (Figure 1).

**Figure 1:**
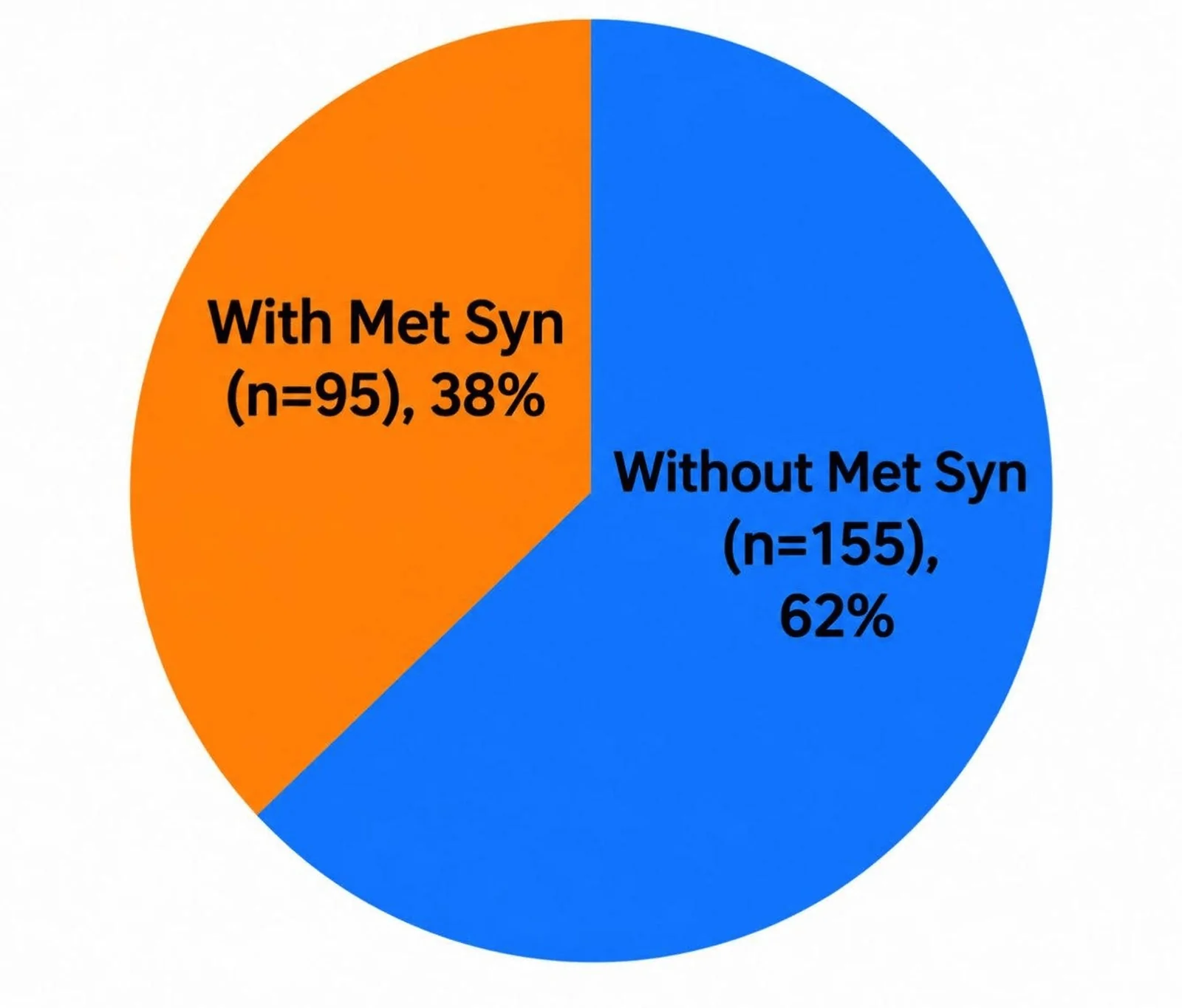
Distribution of metabolic syndrome (38%) among overweight and obese children

Out of 95 participants with metabolic syndrome, 21 (22%) had all defining components of metabolic syndrome. Among the 155 participants who do not have metabolic syndrome, 67 (43.22%) have a WC above the 90th percentile (Figure 2).

**Figure 2:**
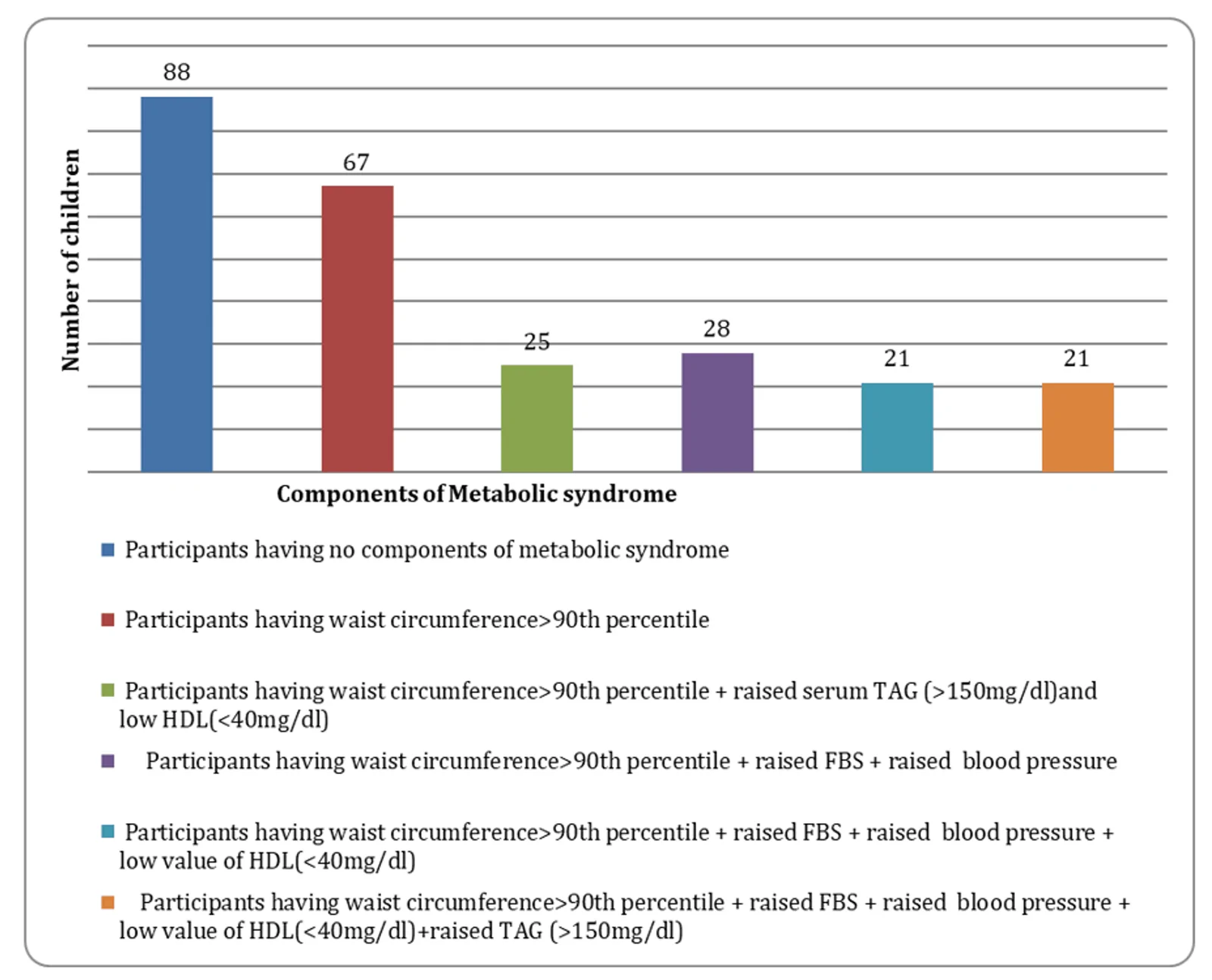
Distribution of children according to the number of metabolic syndrome components FBS, fasting blood sugar; HDL, high-density lipoprotein; TAG, triglyceride.

Table 1 shows that out of 250 participants, 154 (61.6%) were males. There was a higher proportion of females than males in the metabolic syndrome group. Among 154 males, 35.06% had metabolic syndrome, and among 96 females, 42.71% had metabolic syndrome. A total of 177 (70.8%) participants were overweight, and 73 (29.2%) were obese, with the highest percentage of metabolic syndrome among obese children. Those children whose mothers had a history of gestational diabetes mellitus and hypertension during pregnancy have a higher risk of developing metabolic syndrome, with 64.60% and 64.70%, respectively. The mean difference between the groups was significant, with p-values of 0.009 and 0.019, respectively. There was a history of NICU admissions in 42 participants, out of which 26 (61.9%) belonged to the metabolic syndrome group. Upon analyzing the data, 143 (57.2%) participants were exclusively breastfed, and of them, 105 (73.43%) were protected against metabolic syndrome; thus, exclusive breastfeeding for the initial six months of life acts as a protective factor against metabolic syndrome. Children with a family history of obesity are at 28% higher risk of developing metabolic syndrome, and it is also found to be an independent risk factor for metabolic syndrome.

**Table 1:** Sociodemographic history and anthropometry parameters of the study participants p-value <0.05 is significant, obtained using the chi-square test. Data are represented as N (total number of individuals in the category).

Parameter	Total (N=250)	Metabolic syndrome (N=95 (38%))	Non-metabolic syndrome (N=155 (62%))	Chi-square value	p-value
Age group	128 (51%)	48 (37.5%)	80 (62.5%)	0.0278	0.868
<10 years					
>10 years	122 (48%)	47 (38.5%)	75 (61.48%)		
Gender	154 (61.6%)	54 (35.06%)	100 (64.94%)	1.4664	0.226
Male					
Female	96 (38.4%)	41 (42.71%)	55 (57.29%)		
Father education	5 (2%)	2 (40%)	3 (60%)	1.5643	0.457
Below metric					
Metric until intermediate	131 (52.4%)	45 (34.35%)	86 (65.65%)		
Graduate	114 (45.60%)	48 (42.11%)	66 (57.80%)		
Mother education	10 (4%)	5 (50%)	5 (50%)	3.4455	0.179
Below metric					
Metric until intermediate	204 (81.6%)	72 (35.29%)	132 (64.7%)		
Graduate	36 (14.4%)	18 (50%)	18 (50%)		
Family history of obesity	70 (28%)	45 (25%)	135 (75%)	6.7392	<0.001
Obesity present					
Obesity absent	180 (72%)	50 (71.43%)	20 (28.57%)		
Family history of diabetes mellitus	69 (27.6%)	44 (63.77%)	25 (36.23%)	46.613	<0.001
Diabetes mellitus present					
Diabetes mellitus absent	181 (72.40%)	51 (28.18%)	130 (71.82%)		
Family history of hypertension	57 (22.8%)	36 (63.16%)	21 (36.84%)	5.5217	<0.001
Hypertension present					
Absent	193 (77.2%)	59 (30.57%)	134 (69.43%)		
Family history of cardiovascular diseases (CVDs)	26 (10.40%)	10 (38.46%)	16 (61.54%)	1.2214	0.959
CVD present					
CVD absent	224 (89.60%)	85 (37.95%)	139 (62.06%)		
Developmental history	236 (94.40%)	85 (36.02%)	151 (63.98%)	7.0342	0.008
Normal					
Abnormal	14 (5.6%)	10 (38.46%)	4 (28.57%)		
Prenatal parameter	22 (8.80%)	14 (63.64%)	8 (36.36%)	26.8579	0.009
Gestational diabetes mellitus (GDM) in pregnancy					
Gestational diabetes mellitus present					
Gestational diabetes mellitus absent	228 (91.20%)	81 (35.53%)	147 (64.47%)		
Gestational hypertension in pregnancy	17 (6.80%)	11 (64.70%)	6 (35.29%)	19.8349	0.019
Gestational hypertension present					
Gestational hypertension absent	233 (93.20%)	84 (36.50%)	149 (63.95%)		
Neonatal period	42 (16.80%)	26 (61.90%)	16 (38.10%)	12.2439	<0.001
H/o NICU admission present					
H/o NICU admission absent	208 (83.20%)	69 (33.17%)	139 (66.83%)		
Exclusive breastfeeding present	143 (57.20%)	38 (26.57%)	105 (73.43%)	18.5161	<0.001
Exclusive breastfeeding absent	107 (42.80%)	57 (53.27%)	50 (46.73%)		
Birth weight >2.5 kg	191 (76.4%)	55 (22.80%)	136 (71.20%)	29.1016	<0.001
Birth weight <2.5 kg	59 (23.6%)	40 (67.80%)	19 (32.20%)		

Upon analyzing the dietary history and lifestyle as shown in Table 2, it has been found that children who consume a diet with more calories than required and excessive junk food are more prone to developing metabolic syndrome. Lack of physical activity and screen time of more than two hours also contribute. Children with increased screen time are at three times the risk of developing metabolic syndrome. It has been found that obese children have more clinical signs such as acanthosis nigricans, central fat deposition, and strike Albicans than overweight children.

**Table 2:** Distribution of dietary and clinical parameter with metabolic syndrome p-value <0.05 is significant, obtained using the Pearson chi-square test.

Parameter	Total (N=250)	Metabolic syndrome present (N=95)	Metabolic syndrome absent (N=155)	Chi-square value	p-value
Type of diet	171 (68.4%)	71 (41.52%)	100 (58.48%)	2.8467	0.092
Mixed					
Veg	79 (31.6%)	24 (30.38%)	55 (69.62%)		
Calories consumed	57 (22.80%)	46 (80.70%)	11 (19.30%)	59.1167	<0.001
High					
As per required	176 (70.40%)	42 (23.86%)	134 (76.14%)		
Low	17 (6.8%)	7 (41.18%)	10 (58.82%)		
Excess junk food	34 (13.6%)	29 (85.29%)	5 (14.71%)	42.8851	<0.001
Yes					
No	216 (86.4%)	66 (30.56%)	150 (69.44%)		
Physical activity >1 h	95 (38%)	18 (18.95%)	77 (81.05%)	46.4949	<0.001
Present					
Absent	155 (62%)	77 (49.68%)	78 (50.32%)		
Screen time	92 (36.80%)	58 (63.04%)	34 (36.96%)	38.7512	<0.001
>2 h					
<2 h	155 (63.20%)	37 (23.42%)	121 (76.58%)		
Clinical parameters	107 (42.80%)	98 (91.59%)	9 (8.41%)	6.7732	<0.001
Fat deposition					
Present					
Absent	143 (57.20%)	57 (39.86%)	86 (60.24%)		
Acanthosis nigricans	52 (20.80%)	41 (78.85%)	11 (21.15%)	46.4949	<0.001
Present					
Absent	198 (79.20%)	54 (27.27%)	144 (72.73%)		
Striae albicans	70 (28%)	56 (80%)	14 (20%)	72.7929	<0.001
Present					
Absent	180 (72%)	95 (38%)	155 (62%)		

Table 3 shows that regression analysis was performed to remove the confounding factors. It was found that there is an independent association between metabolic syndrome and parameters such as family history of obesity, excess junk food, abnormal developmental history, physical activity <1 h, screen time >2 h, and birth weight <2.5 kg.

**Table 3:** Association of various parameters with metabolic syndrome on logistic regression z-value >1.96 and p<0.05 indicate statistical significance, obtained against logistic regression.

Parameter	p-value	z-value	Adjusted odds ratio	95% CI lower limit	95% CI upper limit
Family history of obesity	0.002	3.11	1.7	1.22	2.48
Excess junk food	0.002	3.14	1.58	1.18	2.11
Development history abnormal	<0.001	3.62	2.07	1.39	3.08
Physical activity <1 h	0.023	2.28	1.67	1.07	2.59
Screen time >2 h	0.049	1.97	1.44	1.01	2.07
Birth weight <2.5 kg	0.009	2.61	1.43	1.09	1.88

## Discussion

Metabolic syndrome represents an escalating pediatric health concern that predisposes children to severe cardiometabolic outcomes, including cardiovascular disease and stroke. The interplay of these metabolic risk factors exerts a synergistic effect that leads to an increase in prevalence rates. Consequently, our findings raised an urgent clinical concern: metabolic syndrome can no longer be regarded strictly as an adult-onset pathology.

In our study, we have found that the prevalence of metabolic syndrome among overweight and obese children is 38%, while in a study carried out in Jammu and Kashmir, the prevalence of metabolic syndrome was 3.8% (boys: 3.9%, girls: 3.8%) and the prevalence of obesity was 9.9% (boys: 9.9%, girls: 10.6%) among the studied children [11]. In a study carried out in Himachal Pradesh, the prevalence of metabolic syndrome was 3% [19]. This can be due to population and geographical differences. Even the study conducted in Korea estimates that the overall weighted prevalence of metabolic syndrome in adolescents was 13.0% [20]. These studies were carried out in the pre-COVID era; therefore, this also contributes to differences in findings.

Our findings indicate that children exposed to more than one hour of screen time per day exhibited an approximately three-fold higher risk of developing metabolic syndrome than those with less than one hour of daily exposure. In alignment with IAP guidelines, a threshold of >1 hour per day was adopted [21]. Discrepancies across international literature may stem from variations in defined thresholds, as India implements a more conservative cut-off relative to other nations.

In our study, we found a significant positive correlation between metabolic syndrome and screen time, consistent with evidence from China demonstrating elevated metabolic syndrome rates among individuals with high sedentary behavior; specifically, screen time of ≥3 hours per day was associated with a 1.15-fold and 1.14-fold increased risk of abdominal obesity and metabolic syndrome, respectively. That study noted that sedentary behavior and moderate-to-vigorous physical activity exert distinct, independent influences on abdominal adiposity compartments and metabolic risk profiles [21-23]. Conversely, investigations from Canada reported no statistically significant relationship between elevated screen time and the prevalence of metabolic syndrome [24-26].

In our study, sedentary behavior -- defined as less than one hour of physical activity per day -- was significantly linked to abdominal obesity. Abdominal obesity is a well-known risk factor, the most common sign of metabolic syndrome, and a marker of dysfunctional fat tissue. We observed a strong inverse relationship between physical activity and both visceral fat buildup and metabolic syndrome, which aligns with earlier studies [25,26]. This suggests that classifying physical inactivity into more detailed levels in future research could help clarify the mechanisms behind these findings.

Our findings indicate that dietary patterns marked by a high intake of snacks and processed foods are associated with an increased risk of metabolic syndrome. Similar results were reported in a study conducted in Korea, where diets high in sodium and sugar were linked to a higher risk of hypertension and abdominal obesity, both of which contribute to the development of metabolic syndrome [26,27].

In our study, a birth weight of less than 2.5 kg emerged as a strong predictor of metabolic syndrome. This finding diverges from several reports indicating that high birth weight, rather than low birth weight, is significantly associated with metabolic syndrome in the general population [28-31]. Conversely, evidence from China identified low birth weight as an independent risk factor for metabolic syndrome, driven by its association with elevated plasma glucose, fasting insulin, serum triglycerides, and blood pressure; notably, each 1-kg increment in birth weight corresponded to a 2.9 mmHg reduction in systolic blood pressure.

A family history of obesity was identified as a critical risk factor that heightens susceptibility to long-term obesity, cardiovascular disease, and metabolic complications. However, relatively few studies have evaluated this variable as a primary determinant of metabolic syndrome [32]. A study conducted in China demonstrated that the risk of pediatric metabolic syndrome was markedly higher in children of mothers with obesity than in those with paternal obesity [33]. Furthermore, parental overweight exhibited a significant positive correlation with elevated risks of metabolic syndrome, abdominal obesity, and reduced HDL-cholesterol levels across both sexes [33].

Our study also evaluated whether the association between developmental delay and metabolic syndrome remained independent of potential confounding factors. Developmental history has not been investigated as a primary risk factor in major studies till now, making this the first report to demonstrate a direct link between developmental milestones and metabolic syndrome. Emerging evidence suggests that early growth failure and developmental delays significantly increase susceptibility to metabolic syndrome. Supporting this concept, a study conducted in the United States showed that early childhood infections and growth failure adversely affect physical and cognitive development while serving as key contributors to adult cardiovascular and metabolic diseases [32].

We observed no significant sex-specific disparities in the prevalence of metabolic syndrome within our study group. In contrast, a systematic review among children and adolescents demonstrated that boys exhibited a higher vulnerability to metabolic syndrome compared with girls, aligning with the broader literature [32,33]. These inconsistent findings may stem from differences in the age groups evaluated and the varied diagnostic criteria used for pediatric metabolic syndrome. Beyond inherent physiological and genetic differences, fully characterizing the biological mechanisms underlying these sex disparities remains challenging [20,23,33,34].

Strengths of the study

Key strengths of this study include the systematic evaluation of overweight and obese children attending a tertiary care hospital and the use of an age-appropriate definition of metabolic syndrome for children.

It is for the first time specifically study on metabolic syndrome is conducted among the overweight and obese children and that too among tribal predominant of Jharkhand as most of the time this subset of population is neglected, many were of the view that under nutrition is prevalent among tribes. Still, this study also shows the changing trends of increasing obesity along with metabolic syndrome prevalence in the population.

Limitations

It is a hospital-based study. Geographical distribution and food habits influence the outcome, which can vary from place to place. Further, socially undesirable activities such as increased screen time are often underreported by parents. Also, many parents, due to a lack of awareness, consider obesity to be healthy and are unwilling to get regular check-ups and relevant investigations done. Some even fail to follow up on it.

## Conclusions

Metabolic syndrome in children arises from an interplay of both modifiable and non-modifiable determinants. Key contributors identified in this study include poor dietary habits, low birth weight, adverse early developmental milestones, a positive family history of obesity, and contemporary lifestyle factors -- predominantly physical inactivity and excessive screen time.

Instilling balanced nutritional habits and promoting regular physical activity early in life are pivotal in altering this trajectory, significantly curtailing the downstream risk of early-onset cardiovascular morbidity and related metabolic complications. ​Crucially, the findings underscore that addressing pediatric obesity cannot occur in isolation. Because parental body mass index strongly correlates with childhood adiposity and subsequent cardiometabolic risk, preventive strategies must adopt an intergenerational framework. ​A targeted public health policy centered exclusively on early-life obesity -- combining clinical surveillance, school-level behavioral reinforcement, and family-centered lifestyle modifications -- is essential to arrest the rising burden of adult cardiometabolic disease at its roots.

## References

[1] Malecka-Tendera E, Mazur A (2006). Childhood obesity: a pandemic of the twenty-first century. Int J Obes.

[2] Azevedo PS, Minicucci MF, Zornoff LA (2015). Obesity: a growing multifaceted problem. Arq Bras Cardiol.

[3] Després JP, Lemieux I (2006). Abdominal obesity and metabolic syndrome. Nature.

[4] Mottillo S, Filion KB, Genest J (2010). The metabolic syndrome and cardiovascular risk: a systematic review and meta-analysis. J Am Coll Cardiol.

[5] Grundy SM (2012). Pre-diabetes, metabolic syndrome, and cardiovascular risk. J Am Coll Cardiol.

[6] Al-Hamad D, Raman V (2017). Metabolic syndrome in children and adolescents. Transl Pediatr.

[7] Dejavitte RA, Enes CC, Nucci LB (2020). Prevalence of metabolic syndrome and its associated factors in overweight and obese adolescents. J Pediatr Endocrinol Metab.

[8] Cheng X, Guo Q, Ju L (2024). Association between sedentary behavior, screen time and metabolic syndrome among Chinese children and adolescents. BMC Public Health.

[9] Wolf RM, Nagpal M, Magge SN (2021). Diabetes and cardiometabolic risk in South Asian youth: a review. Pediatr Diabetes.

[10] Ramesh S, Abraham RA, Sarna A (2022). Prevalence of metabolic syndrome among adolescents in India: a population-based study. BMC Endocr Disord.

[11] Andrabi SM, Bhat MH, Andrabi SR, Kamili MM, Imran A, Nisar I, Nisar U (2013). Prevalence of metabolic syndrome in 8-18-year-old school-going children of Srinagar city of Kashmir India. Indian J Endocrinol Metab.

[12] Khadilkar V, Shah N (2021). Evaluation of children and adolescents with obesity. Indian J Pediatr.

[13] Pasquali R, Casanueva F, Haluzik M (2020). European Society of Endocrinology clinical practice guideline: endocrine work-up in obesity. Eur J Endocrinol.

[14] Khadilkar VV, Khadilkar AV (2015). Revised Indian Academy of Pediatrics 2015 growth charts for height, weight and body mass index for 5-18-year-old Indian children. Indian J Endocrinol Metab.

[15] Banker A, Bell C, Gupta-Malhotra M, Samuels J (2016). Blood pressure percentile charts to identify high or low blood pressure in children. BMC Pediatr.

[16] Kumar T, Das S, Vinayagamoorthy V, Tripathy SK, Malik A, Kundu S (2025). Prevalence of nonalcoholic fatty liver disease among overweight and obese children from a teaching institution of Jharkhand: a cross-sectional study. J Family Med Prim Care.

[17] Zimmet P, Alberti G, Kaufman F (2007). The metabolic syndrome in children and adolescents. Lancet.

[18] Khadilkar V, Yadav S, Agrawal KK (2015). Revised IAP growth charts for height, weight and body mass index for 5- to 18-year-old Indian children. Indian Pediatr.

[19] Gupta A, Sachdeva A, Mahajan N (2018). Prevalence of pediatric metabolic syndrome and associated risk factors among school-age children of 10-16 years living in district Shimla, Himachal Pradesh, India. Indian J Endocrinol Metab.

[20] You MA, Son YJ (2012). Prevalence of metabolic syndrome and associated risk factors among Korean adolescents: analysis from the Korean national survey. Asia Pac J Public Health.

[21] Gupta P, Shah D, Bedi N (2022). Indian Academy of Pediatrics guidelines on screen time and digital wellness in infants, children and adolescents. Indian Pediatr.

[22] Whitaker KM, Pereira MA, Jacobs DR Jr, Sidney S, Odegaard AO (2017). Sedentary behavior, physical activity, and abdominal adipose tissue deposition. Med Sci Sports Exerc.

[23] Oliveira RG, Guedes DP (2016). Physical activity, sedentary behavior, cardiorespiratory fitness and metabolic syndrome in adolescents: systematic review and meta-analysis of observational evidence. PLoS One.

[24] Vanderloo LM, Keown-Stoneman CD, Sivanesan H (2020). Association of screen time and cardiometabolic risk in school-aged children. Prev Med Rep.

[25] Yin N, Yu X, Wang F (2022). Self-reported sedentary behavior and metabolic syndrome among children aged 6-14 years in Beijing, China. Nutrients.

[26] Mark AE, Janssen I (2008). Relationship between screen time and metabolic syndrome in adolescents. J Public Health.

[27] Hirschler V, Bugna J, Roque M, Gilligan T, Gonzalez C (2008). Does low birth weight predict obesity/overweight and metabolic syndrome in elementary school children?. Arch Med Res.

[28] Ambrosini GL, Huang RC, Mori TA (2010). Dietary patterns and markers for the metabolic syndrome in Australian adolescents. Nutr Metab Cardiovasc Dis.

[29] Hulman S, Kushner H, Katz S, Falkner B (1998). Can cardiovascular risk be predicted by newborn, childhood, and adolescent body size? An examination of longitudinal data in urban African Americans. J Pediatr.

[30] Mi J, Law C, Zhang KL, Osmond C, Stein C, Barker D (2000). Effects of infant birthweight and maternal body mass index in pregnancy on components of the insulin resistance syndrome in China. Ann Intern Med.

[31] Guerrero-Romero F, Aradillas-García C, Simental-Mendia LE, Monreal-Escalante E, de la Cruz Mendoza E, Rodríguez-Moran M (2010). Birth weight, family history of diabetes, and metabolic syndrome in children and adolescents. J Pediatr.

[32] DeBoer MD, Lima AA, Oría RB, Scharf RJ, Moore SR, Luna MA, Guerrant RL (2012). Early childhood growth failure and the developmental origins of adult disease: do enteric infections and malnutrition increase risk for the metabolic syndrome?. Nutr Rev.

[33] Yang Z, Li Y, Dong B, Gao D, Wen B, Ma J (2020). Relationship between parental overweight and obesity and childhood metabolic syndrome in their offspring: result from a cross-sectional analysis of parent-offspring trios in China. BMJ Open.

[34] Jahangiry L, Aune D, Farhangi MA (2022). Screen time and the risk of metabolic syndrome among children and adolescents: a systematic review and dose-response meta-analysis. Nutr Metab Cardiovasc Dis.

